# Microstructure of the silk fibroin-based hydrogel scaffolds derived from the orb-web spider *Trichonephila clavata*

**DOI:** 10.1186/s42649-024-00096-x

**Published:** 2024-02-10

**Authors:** Yan Sun, Bon-Jin Ku, Myung-Jin Moon

**Affiliations:** Department of Biological Sciences, Dankook University, Cheonan, 31116 Korea

**Keywords:** Microstructure, Hydrogel, Scaffold, Silk, Spider

## Abstract

Due to the unique properties of the silk fibroin (SF) made from silkworm, SF-based hydrogels have recently received significant attention for various biomedical applications. However, research on the SF-based hydrogels isolated from spider silks has been rtricted due to the limited collection and preparation of naïve silk materials. Therefore, this study focused on the microstructural characteristics of hydrogel scaffolds derived from two types of woven silk glands: the major ampullate gland (MAG) and the tubuliform gland (TG), in the orb-web spider *Trichonephila clavate*. We compared these spider glands with those of the silk fibroin (SF) hydrogel scaffold extracted from the cocoon of the insect silkworm *Bombyx mori*. Our FESEM analysis revealed that the SF hydrogel has high porosity, translucency, and a loose upper structure, with attached SF fibers providing stability. The MAG hydrogel displayed even higher porosity, as well as elongated fibrous structures, and improved mechanical properties: while the TG hydrogel showed increased porosity, ridge-like or wall-like structures, and stable biocapacity formed by physical crosslinking. Due to their powerful and versatile microstructural characteristics, the MAG and TG hydrogels can become tailored substrates, very effective for tissue engineering and regenerative medicine applications.

**Supplementary Information:**

The online version contains supplementary material available at 10.1186/s42649-024-00096-x.

## Introduction

Hydrogel is an example of an elastic gel, formed from a polymer compound solution (Demirci & Khademhosseini 2016). It absorbs and retains a large amount of water, which it uses as a dispersion medium. Hydrogel possesses a cross-linked network structure (Tamura et al. 2000). This cross-linking occurs through valence bonds, hydrogen bonds, and other mechanisms, resulting in a polymer electrolyte with a three-dimensional structure (Xu et al. 2007). The abundance of water in the hydrogel allows substances dissolved within it, as well as substances with relative molecular mass to permeate and diffuse (Liu & Fan 2005; Gulrez et al. 2011; Ahmed 2015).

Due to their remarkable structure, hydrogels are ideal scaffold materials in tissue engineering repair (Prince et al. 1995; Teulé et al. 2012). The aqueous surroundings of hydrogels offer protective advantages for both cells and small molecule drugs (Yu et al. 2016). Additionally, hydrogels promote cell adhesion and proliferation, facilitating biological processes (Baroli 2007). Certain hydrogel types even allow for in situ injection, enhancing their versatility (Shin et al. 2019). The inherent stability of hydrogel shapes, coupled with their uncomplicated fabrication process, ensures ease of handling (Chai et al. 2017).

For over 5,000 years, silk has been a highly utilized material as well as a topic of extensive study. Spider silk has become a popular topic in material science during the past decade. Silk protein is a natural protein polymer secreted by arthropods such as insects and spiders (Belbéoch et al. 2021). Both types of natural silks exhibit intensive mechanical properties, including elasticity and tensile strength, as well as exceptional biological adaptability. Spider silk surpasses strong chemical fibers like aramid and nylon (Hardy & Scheibel 2010).

Spiders use epithelial cells to synthesize silk proteins in glands, and finally spin them into fibers through spinning tubes or other ducts, resulting in natural materials with unique functions (Altman et al. 2003; Moon 2018). The fibers consist of multiple spider fibrils formed by β-sheets combined with amorphous regions. The primary amino acids present in spider silk are alanine, glycine, and serine, with alanine providing strength and glycine imparting elasticity to spider silk (Römer & Scheibel 2008; Kiseleva et al. 2020).

Spider silk stands out as a remarkable biopolymer with exceptional chemical, physical, and biological properties (Blamires et al. 2017). Compared to other biopolymers such as collagen, polylactic acid, fibrin, alginate, and gelatin, spider silk possesses superior biomolecular properties (Withanage et al. 2021). It significantly reduces inflammatory responses, acts as a growth scaffold for various cell types, and promotes cell adhesion (Li et al. 2015; Lee et al. 2016; Salehi et al. 2020). In recent times, spider silk has found extensive use in biomaterials such as films, matrices, and hydrogels, leading to numerous applications in biomedicine (Johansson et al. 2015; Stoppel et al. 2017; Borkner et al. 2019).

The golden orb web spider, *Trichonephila clavate,* is a member of the *Trichonephila* genus, the Araneidae family, and the *Nephila* (Kuntner et al. 2019). These spiders have seven distinct types of silk-producing glands (Park & Moon 2014), each responsible for producing silk with specific compositions and mechanical properties to fulfill biological functions (Eisoldt et al. 2011; Sun et al. 2023).

Despite the increasing use of spider silk as a biomaterial, it is difficult to obtain substantial quantities of natural spider silk, and the production of artificial spider silk poses challenges (Bakhshandeh et al. 2021). In this study, we utilized field emission scanning electron microscopy (FESEM) to examine dragline and cocoon silk-derived hydrogel scaffolds produced from the MAG and TG of *T. clavate* spiders. By comparing the microstructure of these scaffolds with that of silk-based ones, we hope to gain valuable insights into the applications of natural spider silk in biomaterials.

## Materials and methods

In this study, we used the experimental orb-web spider Trichonephila clavata, belonging to the family Aranidae: *Nephilidae*. These spiders were adult females collected near Dankook University in Cheonan, Chungcheongnam-do, South Korea, in mid-October 2020, because the spider silk glands are most developed during this period. *Trichonephila*, previously considered a subgenus of *Nephila*, was elevated to the genus level (Kuntner et al. 2019). Captured spiders were kept in wooden frames (40 × 40 × 10 cm) with acrylic panels on the front and rear sides, providing natural lighting. The spiders consume insects and water daily.

To obtain two spider silk gland samples, carbon dioxide was injected into a sealed vial containing an adult female spider. After 30 s, the spider was anesthetized and dissected under a light microscope. We repeated this procedure with a second adult female spider. The spiders were then placed in a spider’s Ringer solution which consisted of 160 mM NaCl, 7.5 mM KCl, 4 mM CaCl_2_, 1 mM MgCl_2_, 4 mM NaHCO_3_, and 20 mM glucose, with a pH of 7.40 (Moon 2018; Moon & Tillinghast 2020). The major ampullate glands (MAG) and tubular gland (TG) were carefully removed. The glands were ground with dissecting forceps to fully collect the spider silk protein droplets. The collected liquid was transferred to a 2 mL centrifugal tube, centrifuged at 3000 rpm for 30 s, and the supernatant was refrigerated at 4℃.

To prepare the silk fibroin (SF) solution for hydrogel production, cocoons weighing 150 g were cut into small pieces and subjected to degumming three times in 0.05% (w/w) Na_2_CO_3_ solution at 100 °C for 30 min. The fibers were thoroughly rinsed and dried in an oven. The extracted silk fibroin (SF) fibers were then dissolved in a ternary solvent of CaCl_*2*_/CH_*3*_CH_*2*_OH/H_*2*_O (molar ratio 1:2:8) at 70 ± 2 °C for 4 h (Ajisawa 1998). After dialysis and filtration, a SF solution with a concentration of 8wt% was obtained and stored in a refrigerator at 4 °C (Zhang et al. 2015).

The SF hydrogel was formed by pouring the SF solution into a mold, vortexing at 9000 rpm for 7 min, and then incubating the mold at 37 °C for 1–2 h (Yucel et al. 2009). The MAG hydrogel scaffold was created by mixing the spider MAG silk solution with the same silkworm SF solution at a ratio of 1: 3, followed by the MAG hydrogel formation process as the SF hydrogel. The TG hydrogel scaffold was created using the spider TG silk solution, mixed with the silkworm of solution at a ratio of 1:3. The mixed solution was vortexed at 9000 rpm for 7 min and then incubated at 37 °C for 1–2 h.

After preparation, the three hydrogels were placed in a freeze-drying device set at -80 °C at a pressure of 80 mmHg for 48 h to prepare observation samples (FDCF-12012, Oufeilong Company, Shanghai, China). The microstructural analysis of the hydrogels was performed using field emission scanning electron microscopy (FESEM). The samples were coated with a 20 nm layer of platinum-palladium using a Hitachi E-1030 ion sputter coater, and FESEM (S-4300, Hitachi Co., Tokyo, Japan) was used to observe the samples at an accelerating voltage of 5–20 kV (Sun et al. 2020, 2023).

The average porosity as well as the pore size of the three hydrogels were determined by ImageJ (NIH, USA) analysis. Statistical analysis of the pore size and pore wall thickness of the hydrogel was performed through Excel.

## Results

**Fig. 1:**
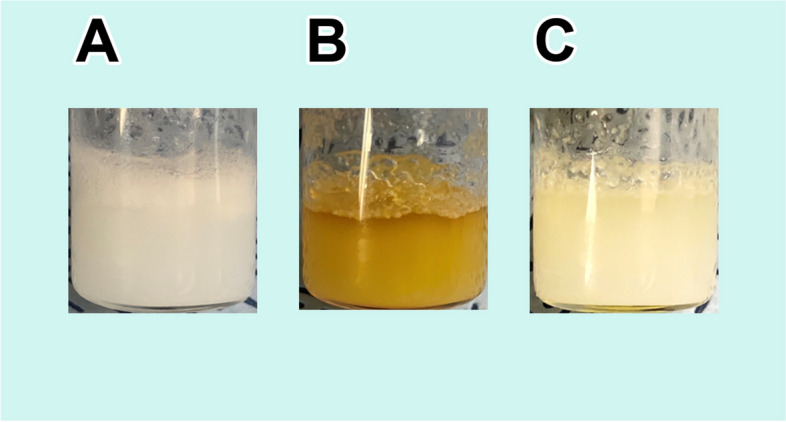
Photomicrograph of 3 types of hydrogels. **A**: The SF hydrogel is translucent, and the structure of the upper layer is looser than that of the lower layer. **B**: The MAG hydrogel is yellow and translucent. **C**: The TG hydrogel is milky white and translucent

Through unaided visual observation, it was evident that the SF hydrogel produced in our experiment possessed a distinctly translucent quality. Notably, the uppermost layer of the hydrogel exhibits a conspicuously more relaxed structure. During its initial stages of formation, the aqueous SF solution went through a vigorous shaking process, resulting in this augmented looseness in its structure (Fig. 1A). In comparison to the SF solution, MAG hydrogels displayed a distinct yellowish translucence due to the presence of yellow protein droplets generated by the MAG of *T. clavata*. Given that the TG of *T. clavata* generated protein droplets with a white hue, the resulting TG hydrogel also appeared milky white and translucent to the unaided eye.

We noted important distinctions in hydrogel structure. Creating the hydrogel through vortexing the protein solution led to a visibly less compact structure in the upper layer of the MAG hydrogels (Fig. 1B). Upon vortexing, the upper layer of the TG hydrogel, like that of the MAG hydrogel, exhibited a looser structure compared to the lower layer, which was discernible to the naked eye (Fig. 1C).

**Fig. 2:**
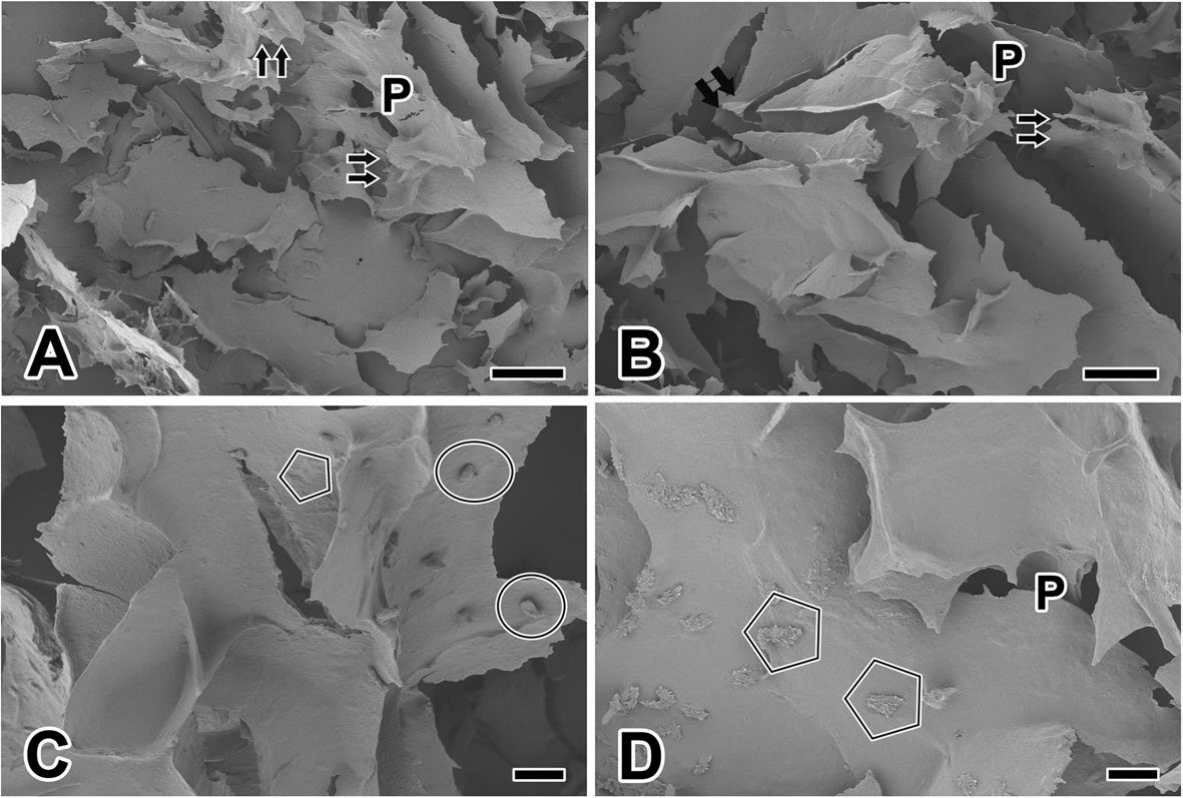
Scanning electron micrographs of the silkmoth’s silk hydrogel scaffolds. **A**, **B**: The hydrogel has a network structure with uneven pores (P) and wall-like structures with sharp fractures (double arrows). The silk scaffold exhibits a porous structure with inconsistent pore wall thickness ranging from 4.5 to 6.5 μm. **C**, **D**: Attached to the hydrogel wall structure are small fragments (circles) approximately 10.0–13.5 μm in length. The wall surface of the hydrogel exhibits a relatively rough texture characterized by closely packed clusters of SF fibers (pentagons). Scale bars indicate 100 μm (**A**, **B**) and 20 μm (**C**, **D**)

Following the freeze-drying of the SF solution at -80 °C, we conducted a detailed examination of the resulting hydrogels using Field Emission Scanning Electron Microscopy (FESEM). The hydrogel revealed a striking high-porosity network structure characterized by ridge-like or wall-like formations. The structure, after drying the hydrogel, was sharp, and the cross-section was uneven (Fig. 2A). Notably, the average porosity of the silk scaffolds registered at an impressive 52.1%. The hydrogel scaffold exhibited a pore-like architecture with an approximate diameter of 59.23 ± 3.70 μm and average thickness of the hole wall is 5.49 ± 0.55 μm (Fig. 2B).

At higher magnification, we discerned block-like structures, measuring approximately 10.0 ~ 13.5 μm in length, adhering to the hydrogel wall. We interpreted them as remnants of small hydrogel fragments that detached during the drying process (Fig. 2C). Additionally, when observed at a higher magnification level, the surface of the hydrogel wall exhibited a rough texture, marked by clusters of SF fibers aggregated together. This type of texture augments surface friction and promotes cell adhesion during cell culture (Fig. 2D).

**Fig. 3:**
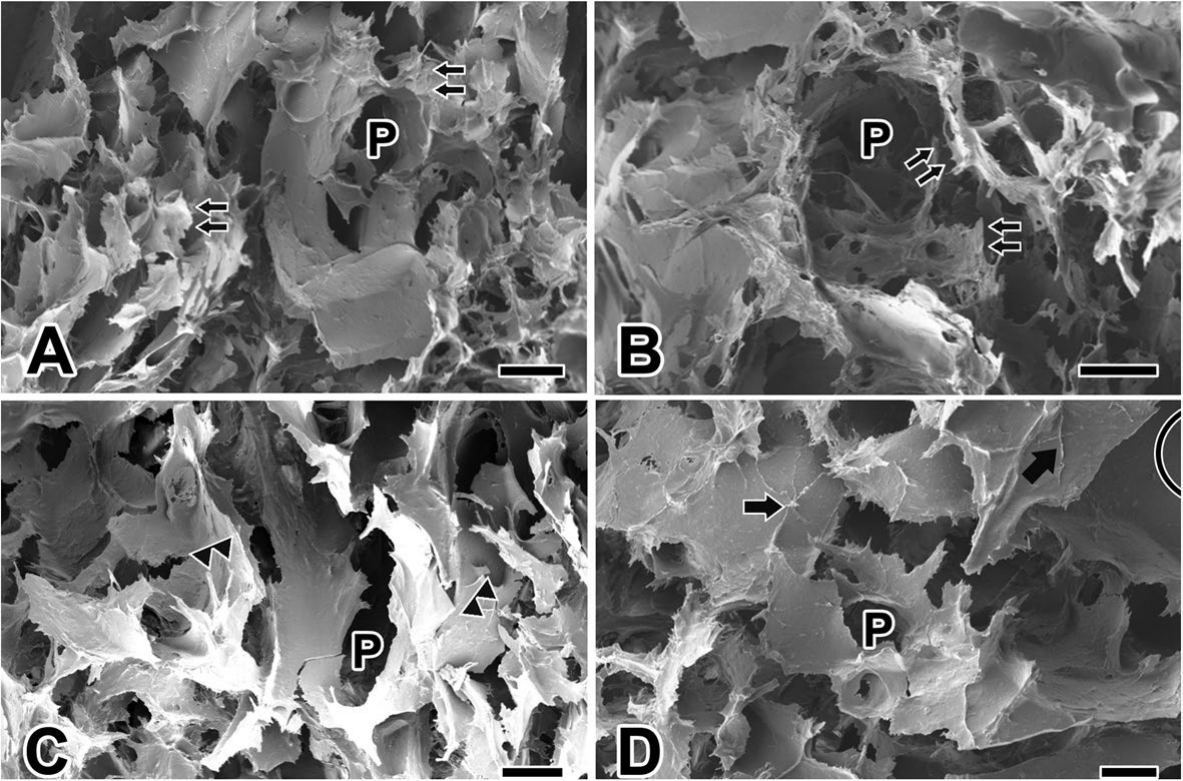
Scanning electron micrographs of the spider’s MAG hydrogel scaffolds. **A**, **B**: The MAG hydrogel has a network structure with uneven pores (P) and sharp cracks (double arrows). Comparing to silkmoth’s SF hydrogel, the pore structure shows greater uniformity in size, and even distribution of the pore walls. **C**, **D**: The average porosity increased by 1.2 times compared with SF hydrogel scaffold. The surface of the hydrogel wall shows a relatively rough texture (arrows), with elongated fiber structures (double arrowheads) attached to the wall. Scale bars represent 100 μm (**A**, **B**), 50 μm (**C**), and 20 μm (**D**), respectively

Similarly, following the freeze-drying process at -80 °C, we employed FESEM to examine the morphological features of MAG hydrogels, created by combining spider MAG protein solution and SF hydrogel at a volume ratio of 1:3. The microstructure of these hydrogels also revealed a network configuration characterized by heightened porosity and the presence of ridge or wall-like structures (Fig. 3A).

Importantly, the fractured appearance of the MAG hydrogel after drying was sharper, while the cross-section appeared flatter in comparison to that of the SF hydrogel. This architectural design promotes efficient material exchange and functions as an excellent conduit for nutrient transport in the context of in vitro cell culture, providing cells with a medium characterized by increased porosity. In comparison to SF gels, the pore structure of MAG hydrogels exhibited greater uniformity in size, with pores distributed more evenly across the pore wall structure (Fig. 3B).

Notably, the average porosity of the MAG hydrogel scaffold stood at 62.6%, which is 1.2 times that of the SF hydrogel scaffold. This elevated porosity enhances nutrient transport and facilitates intercellular nutrient exchange. The diameter of the pore-like structures on the hydrogel scaffold measured approximately 48.12 ± 4.51 μm. However, MAG hydrogels featured thicker pore walls, average thickness of the hole wall is 4.38 ± 0.48 μm, resulting in enhanced mechanical properties relative to SF hydrogels (Fig. 3C).

Upon examination under high magnification, the wall surface of the MAG hydrogel exhibited a rough texture. When scrutinizing the hydrogel wall through high magnification FESEM, slender fiber structures adhering to the hydrogel wall became apparent, with a fiber length measuring typically average 20.1 μm. This structural feature increases surface friction and facilitates cell adhesion during cell culture (Fig. 3D).

**Fig. 4:**
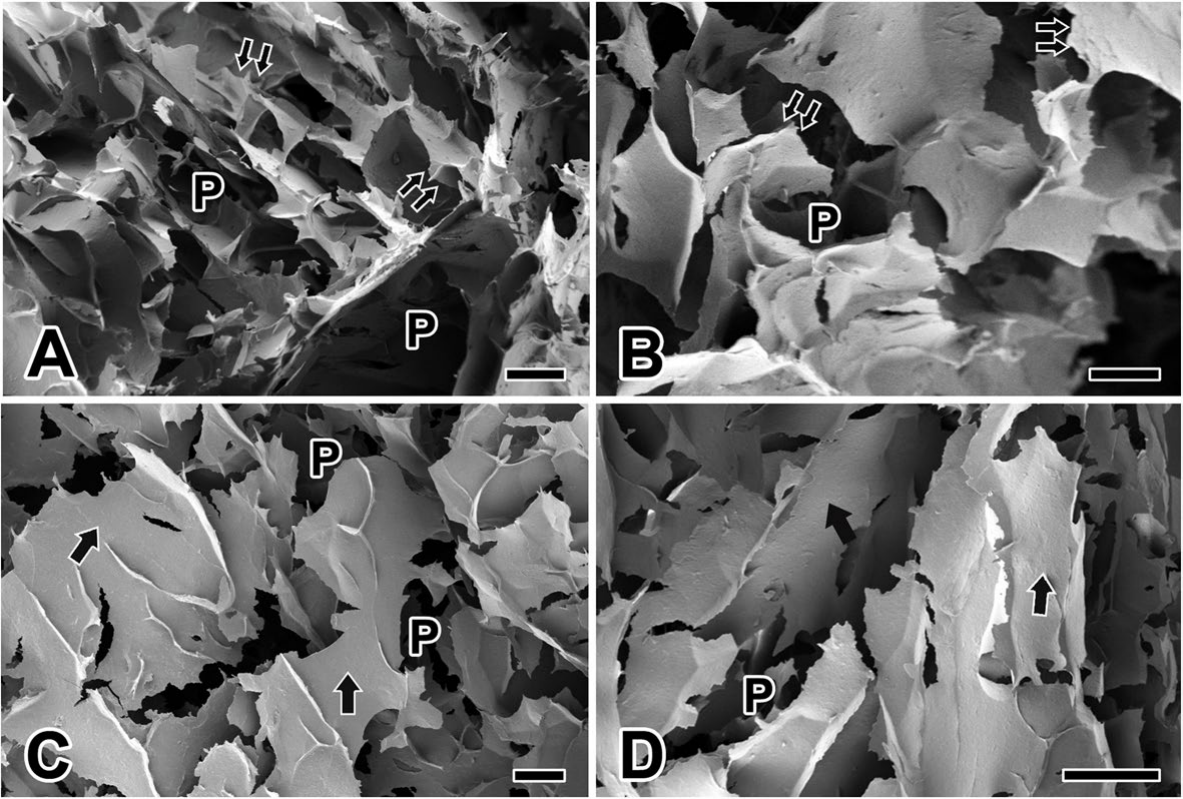
Scanning electron micrographs of the spider’s TG hydrogel scaffolds. **A**, **B**: The TG hydrogel exhibits a network structure characterized by high porosity. The sizes of the pores (P) are not uniform, with ridge or wall-like structures, and breaks in the dry hydrogel wall (double arrows) shows a relatively smooth profile. **C**, **D**: The average porosity of the TG hydrogel scaffold is 52.7%. Compared with other types of hydrogels, pores within TG hydrogels exhibit a more dispersed arrangement lacking the regular shapes. The wall appears smooth (arrows) without any attached fibrous structure. All scale bar indicates 100 μm

We also conducted FESEM analysis to examine the morphological characteristics of freeze-dried TG hydrogels, which were created by blending spider TG protein solution and SF at a volume ratio of 1:3. In comparison to SF, the TG hydrogel manifested a network structure with elevated porosity, featuring ridge or wall-like formations (Fig. 4A). Importantly, fracture appearance of the dried hydrogel wall appeared less sharp than that of the other two hydrogels, and the cross-section exhibited a rather smooth profile. Moreover, in contrast to the MAG hydrogel, the pores in the TG hydrogel appeared more dispersed, lacking the regular shape seen in MAG hydrogels and displaying non-uniform sizes (Fig. 4B).

The average porosity of the TG hydrogel scaffold amounted to 52.70%, positioning it between the porosity values of SF and MAG hydrogels. The pore-like structures on the TG hydrogel scaffold measure 52.0 ~ 54.0 μm in diameter. The thickness of the pore walls ranges from 1.50 to 3.50 μm (Fig. 4C), making TG the thinnest among the three hydrogels and reflecting the weakest relative mechanical properties. Upon closer examination using high-magnification FESEM, the hydrogel wall appeared smooth and devoid of any attached fibrous structures (Fig. 4D).

**Fig. 5:**
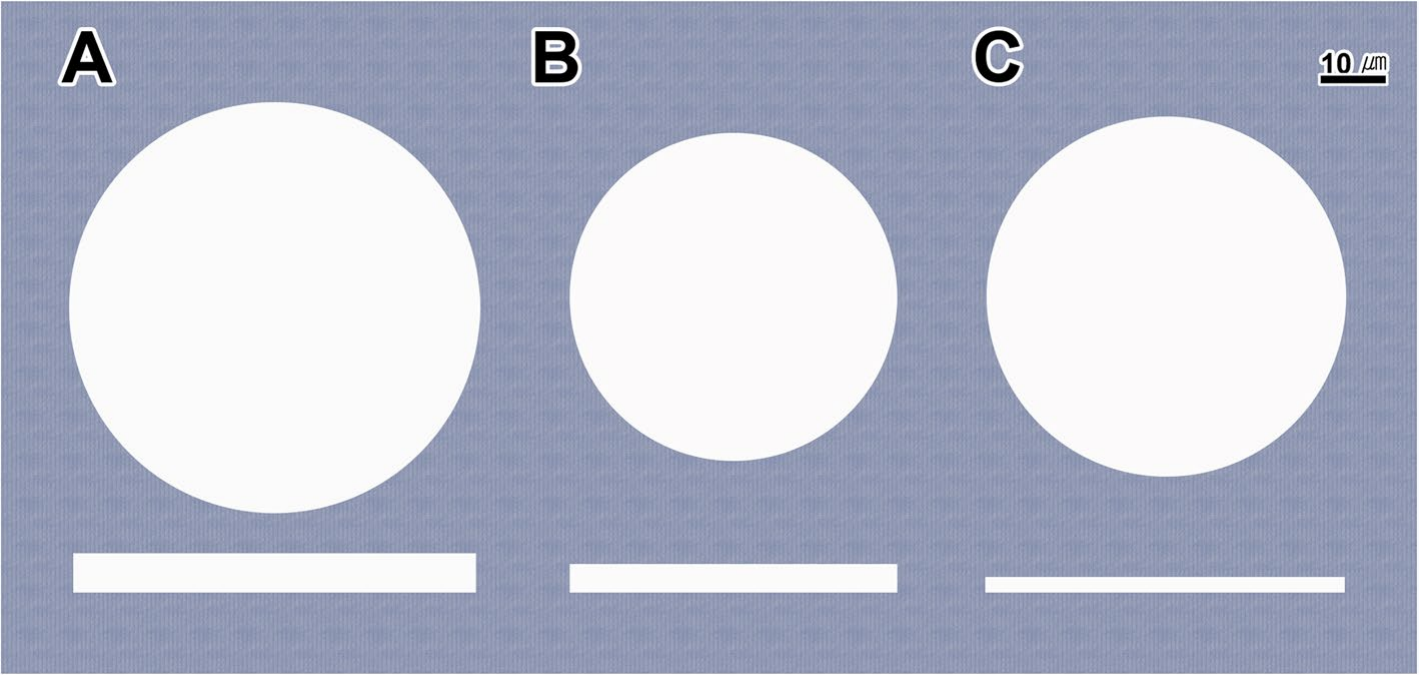
Schematic diagram of the pore wall structure of the hydrogels. Average diameter of each scaffold is SF hydrogel (59.2 μm, **A**), MAG hydrogel (48.1 μm, **B**), and TG hydrogel (52.8 μm, **C**). Average wall-thickness of each scaffold is SF hydrogel (5.5 μm, **A**), MAG hydrogel (4.2 μm, **B**), and TG hydrogel (2.3 μm, **C**), respectively

Following examination of the three hydrogels using Field Emission Scanning Electron Microscopy (FESEM), it became possible to formulate a simplified model diagram based on their distinctive qualities. This diagram demonstrates the pore characteristics of these hydrogels (Fig. 5). The SF hydrogel scaffold displays a porous structure with pore diameters measuring 59.23 μm and pore wall thicknesses spanning from approximately 4.5 to 6.5 μm (Fig. 5A).

Conversely, the MAG hydrogel scaffold featured pore-like structures with a diameter of 48.12 μm, slightly smaller than that of the SF hydrogel, constituting roughly 80% of its pore diameter. Notably, the MAG hydrogels exhibited thicker pore walls within the range of 3.50 to 5.00 μm, resulting in heightened mechanical properties on a relative scale (Fig. 5B).

As for the TG hydrogel scaffold, the diameter of its pore-like structures measured around 53 μm, than that of the SF hydrogel yet approximately 1.1 times greater than that of the MAG hydrogel. The pore walls of the TG hydrogel ranged in thickness 1.50 to 3.50 μm, rendering it the thinnest among the three hydrogels. Relative to the two other hydrogels, TG displayed the least robust mechanical properties (Fig. 5C).

FESEM imaging of the lyophilized hydrogel revealed the fibril structure, and the crosslinked fibril network confirmed the successful physical crosslinking between silk proteins and spider silk proteins, resulting in the formation of the hydrogel network. Among the three hydrogels, the MAG hydrogel had the smallest pore size, while the SF hydrogel had the largest average pore size (Fig. 5).

## Discussion

Hydrogels are three-dimensional network polymers with high water content (more than 95% w/w) and high swelling rates (Rammensee et al. 2006; Yan et al. 2006). Able to absorb and retain a large amount of water, hydrogels utilize it as a dispersion medium. Hydrogels possess a cross-linked network structure. Achieved through valence bonds, hydrogen bonds, and other interactions, their cross-linking result in polymer electrolyte characteristics and a three-dimensional structure (Kopeček 2007; Kabiri et al. 2011).

Fibroin hydrogels form when silk fibroin (SF) molecules combine through non-covalent bonds, such as hydrogen bonds, hydrophobic group interactions, electrostatic interactions, ionic interactions, and chain entanglements. These physically cross-linked formulations make SF hydrogels highly biocompatible and biodegradable, raising their potential for various applications in the biomedical field (Zheng & Zuo 2021).

Pore size and porosity are fundamental parameters used to assess the structural characteristics of hydrogels. Porosity, which refers to the proportion of empty space within a solid material is an important material-independent morphological property for bone tissue formation (Kuboki et al. 1998). A heightened level of porosity fosters cellular proliferation by easing the passage of oxygen and nutrients through larger pore spaces. Conversely, scaffolds featuring lower porosity can enhance cellular functions like osteogenic growth, while retarding cell proliferation and aggregation (Takahashi & Tabata 2004).

Our analysis of SF hydrogels unveiled a robustly porous network structure with a ridge- or wall-like formation facilitating efficient material exchange and nutrient transport. Notably, SF fibers adhered to the walls of the hydrogel, thus bolstering its structural stability. The average porosity of SF hydrogel constituted 52.1%, rendering it translucent with a rather loose upper structure. Similarly, MAG hydrogels exhibited a network structure with even higher porosity and a ridge- or wall-like configuration, actively promoting efficient material exchange and nutrient transport during in vitro cell culture (Karageorgiou & Kaplan 2005).

Additionally, we identified elongated fibrous structures adhering to the hydrogel walls, further fortifying their structural integrity. MAG hydrogel assumes a yellow translucent appearance and a loose upper structure, with an average porosity 1.20 times higher than that of SF hydrogels, suggesting enhanced nutrient transport (Takahashi & Tabata 2004).

Distinctively, TG hydrogels featured elevated porosity levels and unique ridge- or wall-like structures. Unlike SF and MAG hydrogels, TG hydrogels did not host attached fibrous structures on their walls. They presented as milky and translucent, boasting unique pore diameters and thin pore walls, thereby yielding distinctive mechanical properties. These findings align with those reported by Liu et al. (2019), reaffirming successful chemical cross-linking between silk and spider silk proteins, which validates the formation of a robust hydrogel network.

Our FESEM analysis has highlighted the morphological traits of SF, MAG, and TG hydrogels. SF hydrogels, characterized by attached SF fibers, exhibit considerable porosity, while MAG hydrogels show even higher porosity levels, elongated fiber structures, and superior mechanical attributes. TG hydrogels, on the other hand, exhibit augmented porosity coupled with a distinctive ridge- or wall-like structure, signifying successful chemical cross-linking. In the context of cell culture, a study by Yuan et al. (1999) suggested that the roughness and high porosity of MAG hydrogels render them more conducive to cellular proliferation.

Nevertheless, the selection of a hydrogel should be informed by the specific requisites of distinct cellular tissues in both in vitro and in vivo settings (Zou et al. 2022). Our analysis of the morphological properties of these hydrogels can inform their applications in tissue engineering, drug delivery, and regenerative medicine. Subsequent research can build on our findings to further optimize hydrogel performance and expand its practical applications.

SF hydrogels fall into two primary categories of porosity: macropores and nanopores (Isobe et al. 2018). Macroporous SF hydrogels feature larger pore sizes that are discernible to the naked eye or under an optical microscope, typically ranging from tens to hundreds of microns. Their porosity values typically span from 50 to 90%. Nanoporous SF hydrogels possess much smaller pore sizes, usually less than 100 nm (Ak et al. 2013; Isobe et al. 2018).

The specific pore characteristics of SF hydrogels assume a pivotal role in determining their suitability for various applications. Consequently, in line with the findings of Karageorgiou and Kaplan (2005), the hydrogel acquired in this study displayed macroporous attributes, facilitating the unhindered flow of nutrients, oxygen, and cells within the hydrogel matrix. Importantly, the smaller pore size of MAG hydrogels may constrain cellular penetration and migration within the hydrogels. However, by adjusting the MAG protein-to-SF ratio, hydrogels with varying pore sizes might be tailored to meet specific requirements.

Moreover, the thicker pore walls observed in MAG hydrogels enhance their mechanical properties, rendering them suitable for applications where mechanical strength is critical. Conversely, the augmented porosity in TG hydrogels, as demonstrated by Isobe et al. (2018), promises to facilitate efficient material exchange and nutrient transport.

Large-pore silk fibroin hydrogels derived from spider silk proteins exhibit distinctive pore characteristics, wall thickness, and porosity, making them well-suited for loading and controlling the release of therapeutic agents such as growth factors, proteins, and drugs, (Schacht et al. 2015). These hydrogels hold substantial potential as delivery systems for achieving localized and sustained drug release in applications within the domains of tissue engineering and regenerative medicine.

Physical crosslinking involves the transformation of SF molecules from a random coil structure to a β-sheet conformation by manipulating specific physical factors, resulting in further aggregation and the formation of a hydrogel with a three-dimensional network structure (Koh et al. 2015; Aigner et al. 2018). The β-sheet content within the SF solution significantly impacts pore size, distribution, and mechanical properties (Kim et al. 2004; Vepari & Kaplan 2007; Slotta et al. 2008).

The looseness of the structure in the upper layer of SF hydrogel may affect its mechanical properties, limiting its applicability in scenarios demanding a more uniform structure (Widhe et al. 2016). In contrast, the thicker pore walls of MAG hydrogels contribute to their enhanced mechanical properties, rendering them suitable for applications where mechanical strength is paramount. Moreover, the ridge-like or wall-like structure of MAG hydrogels provides efficient nutrient transport channels advantageous for in vitro cell culture. However, compared to SF and MAG hydrogels, TG hydrogels exhibit weak and thin pore walls, which limits their application whenever higher mechanical strength is required.

The β-sheet content in the SF solution influences pore size, distribution, and mechanical characteristics (Koh et al. 2015). On the other hand, spider silk proteins self-assemble into β-sheet-rich nanofibers through the nucleation-aggregation mechanism to achieve gelation, with concentration playing a pivotal role in this process (Römer & Scheibel 2008; Kiseleva et al. 2020).

The distinct morphological characteristics of SF, MAG, and TG hydrogels all offer advantages and disadvantages for various applications. SF hydrogels boast high porosity and stability, albeit with a potentially looser structure. MAG hydrogels exhibit higher porosity, improved mechanical properties, and efficient nutrient transport, though they have smaller pore sizes and a yellow appearance. TG hydrogels feature increased porosity, successful physical crosslinking, and unique morphological attributes, but their mechanical properties are comparatively weaker, and they have a milky white appearance.

The silkworm cocoon, spider egg sac, and spider dragline silk can be effectively employed for chondrocyte culture in vitro (Gellynck et al. 2008). The hydrogel scaffolds generated in this study hold significant potential for future biomedical applications. These scaffolds can be adapted to diverse culture settings, offering tailored substrates with varying porosity and other distinguishing characteristics to best support specific cell types. A comprehensive understanding of these properties is essential when selecting the most suitable hydrogel for specific applications in fields such as tissue engineering, regenerative medicine, and drug delivery.

## Conclusion

Research on SF-based hydrogels isolated from spider silk has been hindered by challenges in the collection and preparation of naive silk materials. Therefore, we focused on the microstructural characteristics of hydrogel scaffolds derived from the major ampullate gland (MAG) and tubuliform gland (TG) of the spider, *T. clavata*. We compared these with a silk fibroin (SF) hydrogel scaffold extracted from the silkworm (*B. mori*) cocoon. FESEM analysis showed that hydrogels derived from spider silk glands exhibited higher porosity, elongated fibrous structure, and improved mechanical properties than SF hydrogels exclusively made from silkworm. Because of their versatile and powerful microstructural characteristics, MAG and TG hydrogels have advantageous applications for tissue engineering and regenerative medicine.

### Supplementary Information

**Additional file 1.**

**Additional file 2.**

## Data Availability

Materials described in the manuscript, including all relevant raw data, will be freely available to any scientist wishing to use them for non-commercial purposes.

## Abbreviations

FESEM: Field emission scanning electron microscopy
MAG: Major ampullate gland
SF: Silk fibroin
TG: Tubuliform gland
