# Supplementary material for: Microstructure of the silk fibroin-based hydrogel scaffolds derived from the orb-web spider Trichonephila clavata
Source: Appl Microsc. 2024 Feb 10;54:3. doi: 10.1186/s42649-024-00096-x (PMC10858014; doi:10.1186/s42649-024-00096-x)
Supplement: Supplementary file 1 — Additional file 1. [file 42649_2024_96_MOESM1_ESM.pdf]

EDITORIAL CERTIFICATE

Date: Jan 11, 2024

Manuscript Author(s): Yan SUN, Bon-Jin KU, and Myung-Jin MOON

Manuscript Title: Microstructure of the Silk Fibroin-based Hydrogel Scaffolds Derived from the Orb-web Spider Trichonephila clavata

To Whom It May Concern:

This letter confirms that the manuscript corresponding to the information detailed above was edited by a professional, native English-speaking editor at Wordvice.
We guarantee 100% language accuracy in the text, as edited and delivered to the author(s) on the date below. We make no claims as to the substantive matter covered by the paper and have not altered the intent or research content drafted by the author(s).
The author(s) may accept or reject any of our comments or suggestions upon receipt of the document we edited. Should you have any questions or concerns, please contact Wordvice at edit@wordvice.com

Sincerely,
Wordvice

Wordvice is a premier international English editing service. Our mission is to help researchers, scholars, students, and professionals reach their full potential through clear communication in their writing. By providing premium English editing services at affordable rates to clients from around the world, Wordvice seeks to tear down language barriers and contribute to the advancement of research and education.

Signature

Kevin Heintz
Managing Editor, Wordvice

Date of Issue
Jan 11, 2024

Contact Wordvice

KOREA	U.S.	JAPAN	TAIWAN	CHINA	TURKEY
edit@essayreview.co.kr	edit@wordvice.com	edit@wordvice.jp	edit@wordvice.com.tw	edit@wordvice.cn	edit@wordvice.com.tr
+82 2-1522-9180	www.wordvice.com	+81 3-4500-8427	+886 2-7738-1133	+86 400-820-0510	+90 0530 390 6216
www.essayreview.co.kr		www.wordvice.jp	www.wordvice.com.tw	www.wordvice.cn	www.wordvice.com.tr
