# Supplementary material for: Microstructure of the silk fibroin-based hydrogel scaffolds derived from the orb-web spider Trichonephila clavata
Source: Appl Microsc. 2024 Feb 10;54:3. doi: 10.1186/s42649-024-00096-x (PMC10858014; doi:10.1186/s42649-024-00096-x)
Supplement: Supplementary file 2 — Additional file 2. [file 42649_2024_96_MOESM2_ESM.docx]

Editor, Ki Woo Kim

Applied Microscopy

January 10, 2024

Manuscript ID: APPM-D-23-00012
Manuscript Title: Microstructure of the Silk Fibroin-based Hydrogel Scaffolds Derived from the Orb-web Spider *Trichonephila clavata*
Authors: Yan Sun; Bon-jin KU; Myung-Jin Moon

Dear Editor Kim:

We appreciate you and the Reviewers for the insightful comments and suggestions on our manuscript. We made a point-to-point reply to the questions raised by the Reviewers, as attached below. By carefully following these suggestions and comments raised by the Reviewers and made a careful revision on the manuscript accordingly. The revisions were marked in red in the revised manuscript. In addition, the English correction was by English editing company (“Wordvice”) and the certificate is at the back of the review response form.

Should you have any further questions, please let me know. Thank you very much for your consideration!

Yours Sincerely,

Myung-Jin Moon

Department of Biological Sciences, Dankook University, Cheonan, 31116, Korea

Tel: +82-41-550-3445; Fax: +82-41-550-3409; Mobile: +82-10-5581-3445

E-mail: moonmj@dankook.ac.kr

**List of Revision Points (2nd Revision) (**Reviewer 1)

| **Page** | **Section** | **Manuscript** | **Revision requested** | **Revised Version** |
| --- | --- | --- | --- | --- |
|  | This research paper is suitable for Applied Microscopy. But found some errers. | | | |
| 5 | Lane 24 | Materials and Methods | Page 5 lane 24: Moon and Tillinghast, 2020 -> Moon & Tillinghast, 2020 | Revised as recommended.   - Page 5 lane 24: Moon and Tillinghast, 2020 🡺 Moon & Tillinghast, 2020 |
| 18 | Lane 1-2 | References | Page 18 in References lane 1: Please confirm the author: A. , U. Demirci  lane 2 Co Inc. -> Co. Inc. | Revised as recommended.   - A. , U. Demirci 🡺 A. U. Demirci - lane 2 Co Inc. 🡺 Co. Inc. |
| 21 | Lane 17 |  | Lane 18 confirm space  3352-3361(2015) -> 3352-3361 (2015) | Revised as recommended:   - Lane 18 confirm space 3352-3361(2015) 🡺 3352-3361 (2015) |
| 21 | Lane 18 |  | Lane 17 CaCl2 -> 2 lower subscript | Revised as recommended:   - Lane 17 CaCl2 -) 🡺CaCl_2_ |
| 21 | Page 21 |  | Lane 21 There are not volume number and page in this journal (Zou et al. 2022). The volume number is 153, page 68-84. | Revised as recommended:   - S. Zou, X. Yao, H. Shao, R. L. Reis, S. C. Kundu, Y. Zhang, Nonmulberry silk fibroin-based biomaterials: Impact on cell behavior regulation and tissue regeneration. Acta Biomaterialia. (2022).🡺 S. Zou, X. Yao, H. Shao, R. L. Reis, S. C. Kundu, Y. Zhang, Nonmulberry silk fibroin-based biomaterials: Impact on cell behavior regulation and tissue regeneration. Acta Biomaterialia. 153, 68-84 (2022). |
|  | - Authors are really appreciated for anonymous reviewer’s precious advice and comments. Thank you very much. | | | |

**List of Revision Points (2nd Revision) (**Reviewer 2)

| **Page** | **Line / Section** | **Manuscript** | **Revision requested** | **Revised Version** |
| --- | --- | --- | --- | --- |
|  | The author responded that the analysis was conducted on twenty samples using ImageJ. Additionally, it is crucial to incorporate the mean and standard deviation of the experimental findings regarding pore size and thickness into the manuscript based on the significant digit. There are several issues concerning the manuscript as follows: | | | |
| 6 | Lane 24 | Materials and Methods | It is crucial to incorporate the mean and standard deviation of the experimental findings regarding pore size and thickness into the manuscript based on the significant digit. | Revised as recommended as follow:   - Added in Materials and Methods section: Statistical analysis of the pore size and pore wall thickness of the hydrogel was performed through Excel. |
| 7 | Lane 23 | Recult | It is crucial to incorporate the mean and standard deviation of the experimental findings regarding pore size and thickness into the manuscript based on the significant digit. | The results were calculated through the standard deviation formula in Excel software, and the following modifications were made:   - The hydrogel scaffold exhibited a pore-like architecture with an approximate diameter of 59.23 μm and pore wall thickness spanning from 4.5 to 6.5 μm (Fig. 2B). 🡺 The hydrogel scaffold exhibited a pore-like architecture with an approximate diameter of 59.23±3.70 μm and average thickness of the hole wall is 5.49 ± 0.55 μm (Fig. 2B). |
| 8 | Lane 19 |  |  | Revised as recommended as follow:   - The diameter of the pore-like structures on the hydrogel scaffold measured approximately 48.12 μm. However, MAG hydrogels featured thicker pore walls, ranging from 3.5 to 5.0 μm, resulting in enhanced mechanical properties relative to SF hydrogels (Fig. 3C). 🡺 The diameter of the pore-like structures on the hydrogel scaffold measured approximately 48.12 ± 4.51 μm. However, MAG hydrogels featured thicker pore walls, average thickness of the hole wall is 4.38 ± 0.48 μm, resulting in enhanced mechanical properties relative to SF hydrogels (Fig. 3C). |
| 9 | Lane 10 |  |  | The results were calculated through the standard deviation formula in Excel software, and the following modifications were made:   - The pore-like structures on the TG hydrogel scaffold measure 52.0 ~ 54.0 μm in diameter. The thickness of the pore walls ranges from 1.50 to 3.50 μm (Fig. 4C), making TG the thinnest among the three hydrogels and reflecting the weakest relative mechanical properties. 🡺 The pore-like structures on the TG hydrogel scaffold measure 53.02 ± 4.58 μm in diameter. The thickness of the pore walls is about 2.62 ± 0.47 μm (Fig. 4C), making TG the thinnest among the three hydrogels and reflecting the weakest relative mechanical properties. |
|  |  | | | |
|  | Authors are really appreciated for anonymous reviewer’s precious advice and comments. Thank you very much. | | | |
